# Prostate cancer risk in patients with melanoma: A systematic review and meta‐analysis

**DOI:** 10.1002/cam4.2995

**Published:** 2020-03-16

**Authors:** Prakash Acharya, Mahesh Mathur

**Affiliations:** ^1^ Department of Dermatology College of Medical Sciences Bharatpur Nepal

**Keywords:** cancer, carcinoma, melanoma, prostate, risk

## Abstract

**Background:**

The risk of prostate cancer in melanoma patients has been frequently assessed. However, a comprehensive meta‐analysis specifically examining this association is lacking. Our aim was to quantify the risk of prostate cancer in melanoma patients based on the available evidence.

**Methods:**

A systematic review of the existing literature was performed in PubMed, Scopus, and Cochrane databases by two authors independently. Studies reporting the effect size in the form of standardized incidence ratio (SIR) were used for quantitative analyses.

**Results:**

Of 17 studies included in the review, a total of 15 studies with 282 592 male melanoma patients were used for the analysis. Random‐effects meta‐analysis found a 24% increased risk (SIR = 1.24, 95% confidence interval 1.18 to 1.30) of prostate cancer in melanoma patients compared to the general population, with a prediction interval of 1.05 to 1.45. The risk was consistently significant for various geographical regions and latitude. Heterogeneity was significant (*I* ^2^ = 75%).

**Conclusion:**

These results suggest that the incidence of prostate cancer is significantly higher in patients with melanoma compared to the general population.

## Graphical abstract

Our study findings show that melanoma patients have a 24% higher risk of developing prostate cancer. This could be attributed to various environmental, hormonal and genetic factors. Early diagnosis of prostate cancer in melanoma patients may help in risk stratification.

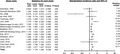

## 1 INTRODUCTION

The worldwide incidence and mortality of cutaneous melanoma continue to rise.^1^ Melanoma is more common in light‐skinned individuals living at lower latitudes.^2^ Prostate cancer (PC) is the second most common cancer in men worldwide, with 3.8% of all cancer‐related deaths in males.^3^ PC has the highest incidences in Oceania, North America, and Europe which overlap with the regions with high melanoma incidence.^4^ While sun exposure is considered a major environmental risk factor for melanoma,^5^ its role in PC is less well‐defined. In fact, most studies conducted previously have reported a protective role of sun exposure in PC,^6^, ^7^ although a few studies contradict this finding.^8^, ^9^ For the diagnosis of melanoma, the initial clinical suspicion through visual and dermoscopic examination is confirmed by histological examination. PC is usually asymptomatic at an early stage and runs an indolent course before being diagnosed by a tissue biopsy.^4^ Early diagnosis of PC can help the clinicians provide appropriate prognostic information to the patients, monitor for disease progression and recommend therapy based on the patient preference and disease prognosis.^10^

Several studies have examined the occurrence of subsequent primary malignancies in melanoma patients.^11^, ^12^, ^13^ Other studies have specifically assessed the risk of PC in melanoma patients.^14^, ^15^, ^16^ A previous meta‐analysis found an increased risk of PC in melanoma patients.^17^ However, multiple newer studies have been published since then.^13^, ^14^, ^18^ Two recent studies from Australia, where the incidence of both melanoma and PC is high, showed an increased risk of PC in melanoma patients.^14^, ^18^ A SEER registry‐based study from the United States showed higher mortality risk in PC patients with a history of melanoma and suggested a serious need to consider PC treatment in such patients.^19^ With this growing interest in the association between the two conditions and possible clinical implications, we conducted this systematic review and meta‐analysis to examine the risk of PC in melanoma patients.

## 2 METHODS

We searched the PubMed, Scopus, and Cochrane databases for the peer‐reviewed published English language articles from inception to 20 July 2019 according to the preferred reporting items for systematic reviews and meta‐analyses (PRISMA) guidelines.^20^ The search terms used were “melanoma” and “prostate cancer” or “prostate carcinoma” and “risk” or “second” or “subsequent” and “epidemiolog*” or “cohort stud*” or “longitudinal stud*” or “case‐control stud*” or “registry”. One of the authors reviewed the title, abstract, and keywords of each resultant study to select potential studies. Full‐text of the resultant studies were reviewed by the authors. References of the retrieved articles were searched for any additional relevant studies. In addition, the list of studies citing the retrieved articles were extracted using Google Scholar and assessed for inclusion. We included cohort studies which reported the risk of developing PC among patients with melanoma and compared the risk with the general population. Studies must have provided the effect size in the form of standardized incidence ratio (SIR) with 95% confidence interval (CI) or the raw data required to calculate the SIR and 95% CI using observed and expected cases and at least adjusted for age. Studies with insufficient data, overlapping cases, reviews, and case reports were excluded. Studies exclusively reporting the cases of ocular melanoma were also excluded.

Two authors extracted and evaluated the data independently. A standardized data‐collection protocol was followed to gather the data from each study and included the primary outcome with the number of observed and expected cases, comparison population, publication year, country of study, study period, study design, adjustment factors and method of diagnosis of melanoma and PC. Both authors assessed the quality of the studies using the Newcastle‐Ottawa scale (NOS) for cohort studies. Any discrepancies between the evaluators regarding the inclusion of the studies were resolved by consensus.

### 2.1 Statistical analysis

The reported SIRs and corresponding 95% CIs were used when provided. For studies which reported the number of observed cases, expected cases, and SIRs, the 95% CI was calculated assuming a Poisson distribution. For studies with fewer than five observed cases, SIRs were not calculated due to the risk of instability.^21^ These studies were included in the qualitative review but were excluded from the meta‐analysis. Considering the differences in study design and baseline participant characteristics, we used the random‐effects model by DerSimonian and Laird.^22^ The 95% prediction interval was calculated to examine the predicted range for the true effect size if a new study is conducted.^23^ Chi‐squared test and *I* ^2^ statistic were used to assess the heterogeneity. *I* ^2^ value greater than 50% was considered to denote significant heterogeneity not attributable to random error.^24^ Subgroup analyses were performed based on study size, data source, geographical region, and latitude. To examine the robustness of the data, a sensitivity analysis was performed by removal of a single study at a time, while observing the impact on the overall effect size. The potential publication bias was assessed visually using the funnel plot and statistically using Egger's test and Begg's rank correlation test.^25^, ^26^ A *P*‐value of less than .05 was considered statistically significant for all analyses. We used Comprehensive Meta‐Analysis software (version 3; Biostat, Inc) for all statistical analyses and generation of graphical plots.

## 3 RESULTS

**FIGURE 1:**
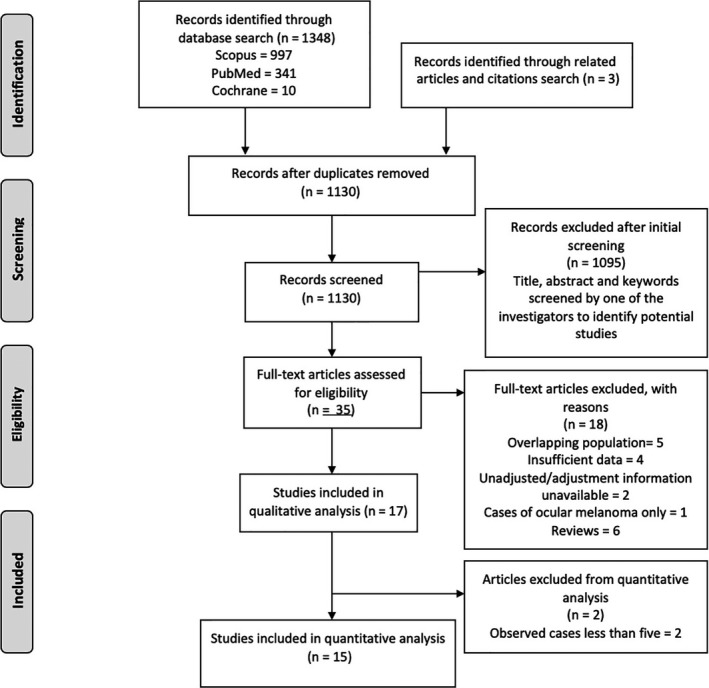
Preferred reporting items for systematic reviews and meta‐analyses flow diagram

The initial search identified 1351 records which were screened according to the inclusion and exclusion criteria. Seventeen studies were finally included in the study of which 15 studies were used for quantitative analysis.^11^, ^12^, ^13^, ^14^, ^15^, ^16^, ^18^, ^27^, ^28^, ^29^, ^30^, ^31^, ^32^, ^33^, ^34^, ^35^, ^36^ Of note, one study did not provide data for age adjustment and reported odd's ratio as the effect size and another study with small sample size also lacked data regarding the adjustment.^37^, ^38^ One study was based on the cases of ocular melanoma only and another study used nonmelanoma patients as control.^39^, ^40^ These four studies were excluded from the review. The study selection process is summarized using the PRISMA flow chart in Figure 1.

### 3.1 Study characteristics

Seventeen studies reported either SIR or the number of observed and expected cases. Two of these studies reported fewer than five observed cases and were excluded from the meta‐analysis.^28^, ^35^ Among 15 studies (282 592 male melanoma patients) included in the meta‐analysis, 12 were registry‐based cohort studies and the remaining three were single‐center cohort studies. Two of these studies did not provide information regarding the number of male melanoma patients.^16^, ^34^ Four studies specifically assessed the risk of developing PC in melanoma patients.^14^, ^15^, ^16^, ^34^ For three studies which did not report the 95% CI for SIRs, the values were calculated using the raw data. Significantly increased risk of PC in melanoma was reported in 10 of these studies. Two studies provided data for in situ melanoma.^11^, ^18^ Diagnostic confirmation for both melanoma and PC was based on International Classification of Diseases codes for registry‐based studies and histology for single‐center cohorts. Quality of the studies examined using NOS ranged from 6 to 8. The study characteristics are summarized in Table 1.

**TABLE 1:** Characteristics of studies reporting risk of prostate cancer in melanoma patients

Study	Country	Study type	Study period	Number of male patients with melanoma	Observed prostate cancer cases	Expected prostate cancer cases	Comparison population	SIR (95% confidence interval)	Adjustments	NOS
Swerdlow, 1995^27^, ^,a^	Denmark	Registry based	1943‐1989	5061	29	NA	General Danish population	0.83 (0.56‐1.20)	Age, sex, and calendar year	7
Bhatia, 1999^28^	USA	Single‐center cohort	1952‐1996	298	2	5.1	General population in SEER registry	Not calculated	Age and sex	6
Schmidt‐Wendtner, 2001^29^	Germany	Single‐center cohort	1977‐1992	2083	23	18	General population of the same state	1.3 (0.8‐1.9)	Age and sex	7
Wu, 2006^30^	USA	Single‐center cohort	1987‐2001	498	8	29.4	General population in SEER registry	0.27 (0.12‐0.54)	Age and sex	6
de Vries, 2007^15^	Netherlands	Registry based	1972‐2002	1420	19	16	General population in the registry	1.16 (0.71‐1.85)	Age, 5‐y period	7
Tuohimaa, 2007^31^, ^,a^	Multinational	Registry based (13 registries from 5 continents)	NA	66 441	1486	1174	General population in the registry	1.27 (1.20‐1.33)	Age, sex, and calendar year	7
Levi, 2008^16^	Switzerland	Registry based (two Swiss registries)	1974‐2005	3346^c^	54	30.6	General population in the registry	1.77 (1.33‐2.3)	Age, sex, site, and calendar year	8
Balamurugan, 2011^11^, ^,b^	USA	Registry based (13 population‐based registries in US)	1992‐2006				General population		Age and sex	6
				In situ melanoma—22 013	906	NA		1.24 (1.16‐1.32)^d^		
				Invasive—41 715	1254	NA		1.15 (1.08‐1.22)^d^		
Bradford, 2011^32^, ^,b^	USA	Registry based (nine population‐based registries in US)	1973‐2006	47 804	2200	1913	General population	1.15 (1.10‐1.20)	Age, sex, and calendar year	6
AIRTUM Working group, 2013^33^	Italy	Registry based	1976‐2010	16 851	267	NA	General population in the registry	1.17 (1.03‐1.32)^d^	Age, geographic area, cancer site, and calendar‐group	7
Kok, 2013^34^	Netherlands	Registry based	1989‐2008	NA	374	NA	General Dutch population	1.39 (1.25‐1.53)^d^	5‐y age and 1‐calender year	7
Toth, 2013^35^	Hungary	Single‐center cohort	2006‐2010	366	1	NA	General Hungarian population	Not calculated	Age and sex	7
Robsahm, 2014^13^	Norway	Registry based	1955‐2008	12 972	445	NA	General Norwegian population	1.26 (1.15‐1.38)	Age and calendar period	7
Jung, 2014^36^	Canada	Registry based	1979‐2009	780	95	NA	General Canadian population	1.2 (1.0‐1.5)	Age, sex, and calendar year	8
Caini, 2016^12^	Italy	Single‐center cohort	2000‐2010	753	6	6.2	General population	0.97 (0.44‐2.17)	Age, sex, calendar year and, country macro‐region	6
Cole‐Clark, 2018^14^	Australia	Registry based	1972‐2008	42 396	2114	1687.82	General population in the registry	1.25 (1.20‐1.31)	Age and calendar period	7
Kimlin, 2018^18^	Australia	Registry based	1982‐2012	21 805 in situ melanoma cases	1264	NA	General population	1.35 (1.28‐1.43)	Age, sex, and calendar year	6

Abbreviations: NA, not available; NOS, Newcastle Ottawa scale; SIR, standardized incidence ratio.

^a,b^ These studies used same data source and may be partially overlapping.

^c^ Separate data for male are not available.

^d^ 95% confidence intervals were calculated for these studies from raw data.

### 3.2 Melanoma and risk of prostate cancer

**FIGURE 2:**
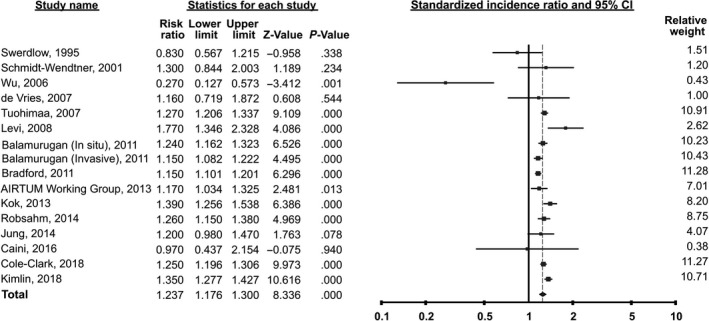
Forest plots showing an increased risk of prostate cancer in melanoma patients (SIR = 1.24, 95% confidence interval 1.18 to 1.30)

The random‐effects meta‐analysis of 15 studies revealed 24% increased risk of prostate cancer in melanoma patients compared to the general population, with SIR of 1.24 (95% CI 1.18 to 1.30, *I* ^2^ = 75%, Figure 2). The 95% prediction interval ranged from 1.05 to 1.45. Sensitivity analysis performed by excluding a single study at a time did not affect the statistical significance of the result. The result remained almost unaffected by removing the two studies of in situ melanoma (SIR = 1.22, 95% CI 1.15 to 1.29).

### 3.3 Subgroup analyses

Subgroup analyses revealed that larger studies (more than 5000 male melanoma patients) showed significantly increased risk (SIR = 1.23, 95% CI 1.17 to 1.28, 8 studies) of PC in melanoma while the smaller studies showed a statistically nonsignificant increased risk (SIR = 1.08, 95% CI 0.76 to 1.54, 6 studies). Similarly, this effect estimate was significant (SIR = 1.24, 95% CI 1.19 to 1.30, 12 studies) for registry‐based studies and nonsignificant (SIR = 0.72, 95% CI 0.28 to 1.85, 3 studies) for single‐center cohort studies. Analyses according to geographical regions and latitude showed statistically significant results in all subgroups. The results of subgroup analyses are summarized in Table 2.

**TABLE 2:** Subgroup analyses for the risk of prostate cancer in melanoma patients

Subgroup	Number of studies	Random effects meta‐analysis SIR (95% CI)	*P* value	*I* ^2^ value
Study location				
Australia	2	1.30 [1.20, 1.40]	**<.001**	78%
Europe	8	1.27 [1.14, 1.42]	**<.001**	55%
North America	4	1.16 [1.07, 1.26]	**<.001**	78%
Latitude				
Low (<45 degree)	7	1.20 [1.13, 1.29]	**<.001**	83%
High (>45 degree)	7	1.29 [1.15, 1.45]	**<.001**	54%
Study type				
Registry‐based	12	1.24 [1.19, 1.30]	**<.001**	73%
Single center cohort	3	0.72 [0.28, 1.85]	.494	84%
Study size				
Large	8	1.23 [1.17, 1.28]	**<.001**	75%
Small	6	1.08 [0.76, 1.54]	.664	78%

The values in bold denote statistically significant findings.

Abbreviations: CI, confidence interval; SIR, standardized incidence ratio.

### 3.4 Publication bias

**FIGURE 3:**
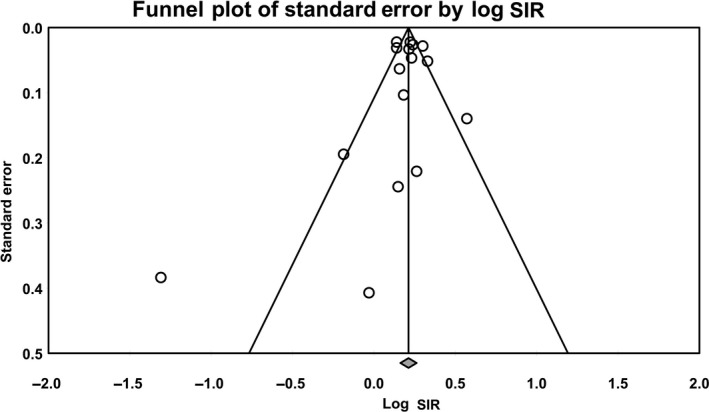
Funnel plot for the risk of prostate cancer in melanoma patients

Visual examination of the funnel plot to assess the publication bias showed symmetry and absence of bias (Figure 3). Begg's rank correlation test (*P* = .18) and Egger's test (*P* = .28) did not show any evidence of publication bias.

## 4 DISCUSSION

This study found that the melanoma patients were at higher risk of developing subsequent PC than the general population. The results in this study were mainly pooled from population‐based registries across various geographical regions. A previous meta‐analysis which assessed the risk of various secondary primary cancers in melanoma patients had also reported an increased risk of PC in patients with melanoma (SIR = 1.25, 95% CI 1.13 to 1.37, 9 studies).^17^ This is similar to the findings of the current study (SIR = 1.24, 95% CI 1.18 to 1.30, 15 studies); however, the number of studies included in the current meta‐analysis were higher and the narrower confidence interval of the effect size in this study provides a more precise estimate. Furthermore, we also found that the elevated risk remained consistent in various subgroup analyses and sensitivity analyses.

The precise pathophysiological mechanism responsible for the increased risk of PC among patients diagnosed with melanoma is currently unknown. Researchers have attempted to explain the association using various hypotheses pertaining to environmental, hormonal, and genetic aspects, among which, the role of sun exposure is the most described one. Exposure to solar radiation, especially intermittent sun exposure and a history of sunburn is considered a leading environmental cause of melanoma.^41^ High incidences of melanoma in fair‐skinned individuals living at low latitudes further highlights the role of sun exposure in melanoma.^2^ A recent study from Australia which showed an increased risk of PC in melanoma patients described sun exposure as the common factor for the development of both melanoma and PC.^14^ This was supported by an earlier study by the authors from the same location which reported an increased risk of PC with increased sun exposure.^8^ In a genetic study, the same authors found an association between melanoma‐associated pigmentary genes (SNPs—rs1805007 and rs4911414) and increased PSA levels (considered a biomarker of PC) in Australia and New Zealand born males.^42^ While this may possibly explain the association in certain geographical regions with high UV exposures where risk of PC is positively related with sun exposure, majority of studies from other regions and a systematic review have shown inverse relation between sun exposure and the risk of PC.^6^, ^7^, ^43^ Studies have attributed this protective role of UV exposure in PC to the formation of vitamin D.^6^, ^43^ Although some studies have reported lowered risk of PC in NMSC and attributed it to the protective effect of sun exposure, the observed cases of PC were still higher than the expected cases for melanoma in those studies.^15^, ^31^ Additionally, Tuohimaa et al^31^ found no significant difference in risk of PC among melanoma patients in sunny countries and less sunny countries. Our current analysis also found similar risks of PC in melanoma patients living at higher and lower latitudes. These inconclusive findings regarding the role of sun exposure and vitamin D in the increased risk of PC in melanoma patients have led to the researchers proposing alternate pathways for the association.^7^, ^34^

Androgen dependency of both melanoma and PC may explain the relationship between the two tumors. The role of androgens in the carcinogenesis is well known. Androgens have also been found to play an important role in the development of melanoma through multiple mechanisms which include increased melanoma cell proliferation, stimulation of telomerase activity, and suppression of the immune system.^40^ In a genetic study, Cooper et al^44^ studied multiple PC risk alleles in patients of European ancestry and found that certain single nucleotide polymorphisms (SNPs) such as rs1512268 and rs5759167 may be associated with increased risk of melanoma in PC patients.

Detection bias is an important factor to consider while analyzing the results of epidemiological studies. Detection of one type of cancer may lead to the increased surveillance for the cancers occurring elsewhere. Similar phenomenon may have played a role for the increased risk of PC in melanoma patients seen across the included studies. Except for the study by Kok et al,^34^ all other studies which reported the period‐wise detection of PC following the diagnosis of melanoma found the highest risk within the first year of diagnosis.^11^, ^14^, ^31^, ^32^ This is attributed to the increased surveillance for other cancers using screening procedures and the increased vigilance of the patients about their health following the diagnosis of melanoma.^14^, ^31^ This seems to be a logical explanation because patients diagnosed with one form of invasive cancer are commonly screened for other health problems and undergo imaging tests for metastasis.

In subgroup analyses, we found that larger studies and registry‐based studies (almost overlapping with each other) showed significantly increased risk while smaller and single‐center cohort studies showed nonsignificant risk. Subgroup analyses also showed higher risk in European studies compared to the studies from Australia and North America; however, the confidence range was comparatively wider for the analysis, which limited the possibility of deriving any conclusions. Although we did not perform a formal analysis due to limited data, we observed that the risk of developing PC was lower for head and neck melanoma compared to the melanomas at other sites.^15^, ^16^, ^18^ Head and neck melanomas, compared to other melanomas, are assumed to be more associated with chronic sun exposure.^15^ Adequate sun exposure, in turn, generally results in sufficient vitamin D levels. If vitamin D is assumed to protect against PC, the lowered risk of PC in head and neck melanomas can be explained.

The limitation of our study include a considerable heterogeneity due to the variation in effect estimates of the included studies, which could be attributed to the baseline differences among included subjects. We attempted to address this limitation through various approaches. First, we performed subgroup analyses according to various categorical variables. We used a random‐effects model for the analysis and also calculated the prediction interval. The role of *I* ^2^ value as a sole measure of heterogeneity has been questioned and prediction interval is recommended to assess heterogeneity in random‐effects meta‐analyses.^23^ Based on the value of the prediction interval, we can be confident that the true SIR in the studies conducted in the future will fall in the approximate range of 1.05‐1.45 in 95% of the population. This can be considered as a strength of our study. Sensitivity analyses were also performed to assess the statistical stability of the results. A large sample population was available for the analysis and most of the included studies used population‐based registries as the data source, reducing the risk of bias encountered in small studies. Moreover no evidence of publication bias was observed based on graphical and statistical tests.

This study based on available evidence showed an increased risk of PC in melanoma patients. However, it is still unclear whether melanoma is a causal risk factor for PC or weather the detection bias resulted in the increased incidence of PC in melanoma patients. Future studies should consider assessing whether the melanoma patients actually benefit from screening for PC.

## CONFLICT OF INTEREST

The authors declare no conflicts of interest.

## AUTHORS’ CONTRIBUTIONS

PA and MM contributed to study design and literature review and analyses.PA contributed to literature search and analyses. MM contributed to manuscript drafting.

## Data Availability

This study analyzes the data from previous studies and the cited studies contain all data reported in this manuscript.
